# Angelman-Like Syndrome: A Genetic Approach to Diagnosis with Illustrative Cases

**DOI:** 10.1155/2016/9790169

**Published:** 2016-01-28

**Authors:** Ho-Ming Luk

**Affiliations:** Clinical Genetic Service, Department of Health, Kowloon, Hong Kong

## Abstract

Epigenetic abnormalities in 15q11-13 imprinted region and* UBE3A* mutation are the two major mechanisms for molecularly confirmed Angelman Syndrome. However, there is 10% of clinically diagnosed Angelman Syndrome remaining test negative. With the advancement of genomic technology like array comparative genomic hybridization and next generation sequencing methods, it is found that some patients of these test negative Angelman-like Syndromes actually have alternative diagnoses. Accurate molecular diagnosis is paramount for genetic counseling and subsequent management. Despite overlapping phenotypes between Angelman and Angelman-like Syndrome, there are some subtle but distinct features which could differentiate them clinically. It would provide important clue during the diagnostic process for clinicians.

## 1. Introduction

Since the first description of Angelman Syndrome (AS) by Dr. Angelman in 1965 [1], there was a great advancement in understanding of its clinical features and molecular genetic mechanism. AS is characterized by distinct facial gestalt, developmental delay, absent speech, ataxic gait, seizure, and paroxysms of laughter [2]. The incidence reported was about 1/12,000 to 1/20,000 [3, 4] without racial predilection. The diagnosis of AS depends on the combination of clinical criteria and molecular and/or cytogenetic testing. The consensus criteria for clinical diagnosis of AS were proposed in 2006 [5] which included a list of core and associated features. However, the clinical manifestations of AS were highly heterogeneous that would overlap with other diseases. Methylation study on 15q11-13 imprinted region would identify 75–80% of AS that included maternal deletion, paternal uniparental disomy (UPD), and imprinting center defect. Further analysis of* UBE3A* gene would further confirm 10% of cases. However, there were still 5–10% of clinically diagnosed AS that would be rendered “test negative.” With the advancement of medical genomic technology like array comparative genomic hybridization (array CGH) and next generation sequencing, it was now known that some patients of these “test negative” Angelman-like Syndromes actually had alternative genetic diagnoses [6–8] which were important for counseling and management.

In this review, we use 4 illustrative cases to provide the overview of some Angelman-like Syndromes and highlight their difference with AS, so as to provide some guidance to clinicians on the diagnostic workup when they encounter such patients in their practice.

## 2. Illustrative Cases

### 2.1. Case  1

**Figure 1:**
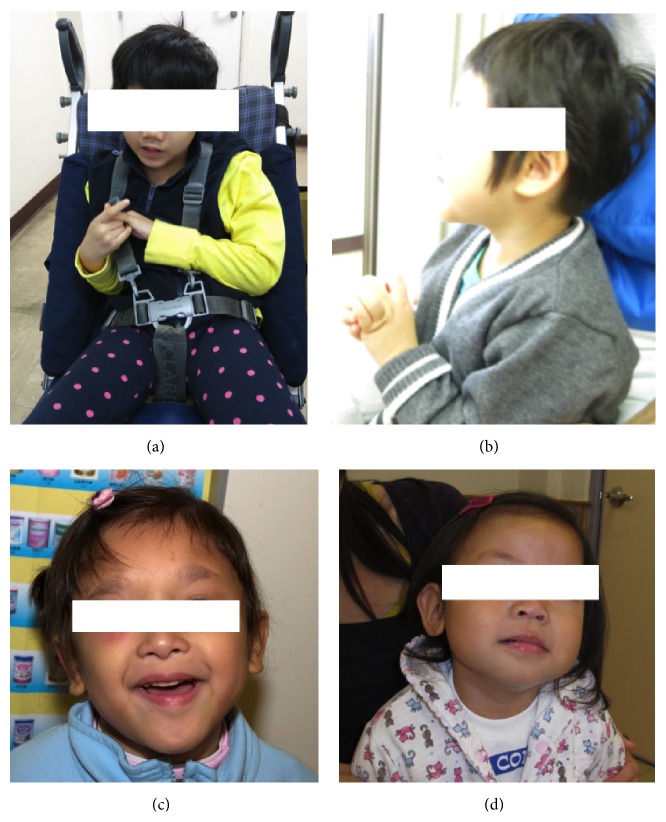
Facial features of different Angelman-like Syndromes in this series. (a) FOXG1 related disease; (b) Rett Syndrome. (c) Mowat-Wilson Syndrome; (d) Phelan-McDermid Syndrome.

A 6-month-old girl was referred to genetic clinic for developmental delay. She was the second child of nonconsanguineous Chinese couple, born at full term with birth weight of 3.83 kg. The perinatal history was unremarkable. She was noted to have microcephaly (head circumference <3th percentile, body weight and body height at 75th percentile) and hypotonia at 3 months of age. Investigations including metabolic screening, muscle enzyme, and computerized tomography of brain were normal. Physical examination at 6 months of age showed microcephaly, flat occiput, right divergent squint, and hypotonia. No syndromal diagnosis could be ascertained at that time and she was regularly followed up in genetic clinic. She had epilepsy since she was 2 years of age and severe global delay at developmental assessment. EEG showed nonspecific background slowing, but no epileptiform abnormalities. Brain Magnetic Resonance Image (MRI) showed mild thinning of corpus callosum without major structural defect. There was no developmental regression, but she developed stereotypical hand movements (Figure 1), bruxism, and occasional outburst of laugher. Based on the craniofacial features like microcephaly, flat occiput, divergent squint, characteristic stereotypical hand movement, and outburst of laughter, Angelman/Rett Syndrome was suspected. However, genetic investigations including methylation-specific multiplex ligation-dependent probe amplification (MS-MLPA) for AS,* UBE3A* gene,* MECP2* gene, and array CGH studies were negative. Based on the MRI findings and early onset of microcephaly,* FOXG1* related disease was suspected.* FOXG1* gene test showed a de novo frameshift pathogenic mutation* FOXG1*{NM_005249.3}:c.[396_397ins26];[=];*FOXG1*{NP_005240.3}:p.[(Gly133Trpfs^*∗*^68)];[=] which confirmed the diagnosis of* FOXG1* related congenital variant of Rett Syndrome.

### 2.2. Case  2

A 10-month-old girl was referred to genetic clinic for global delay. She was the first child of nonconsanguineous Chinese couple, born at 38-week gestation with birth weight of 3.24 kg. Mother had gestational diabetes mellitus that required insulin therapy. She had mild grade bilateral hearing impairment and left divergent squint diagnosed at birth. On follow-up, she was noted to have microbrachycephaly and global developmental delay (Figure 1). Brain MRI, metabolic screening, and array CGH were normal. She had stereotypical handwashing movement since she was 1 year old. There was no clinical or electrical seizure. Based on the craniofacial features like microbrachycephaly, wide mouth, divergent squint, and behavioral phenotype, AS was initially suspected, but the methylation study and* UBE3A* gene test were negative. Subsequently she had bruxism and developmental regression since she was 1 year and 6 months of age with loss of some motor and social skill.* MECP2* study showed de novo nonsense mutation* MECP2*{NM_004992.3}:c.[808C>T];[=];*MECP2*{NP_004983.1}:p.[(Arg270^*∗*^)];[=] that confirmed the diagnosis of Rett Syndrome.

### 2.3. Case  3

A 5-year-old girl was referred to genetic clinic for AS based on the facial dysmorphism. She was the first child of nonconsanguineous Chinese couple, born at full term with birth weight of 2.9 kg. Perinatal history was unremarkable. She was noted to have dysmorphism and cardiac murmur during neonatal period. Echocardiogram showed patent ductus arteriosus and large secundum atrial septal defect. Total corrective operation was done at 1 year of age. Developmental assessment at 2 years of age showed severe grade developmental delay. Stereotypical hand movement, abnormal outburst of laughter, and ataxic gait were developed afterward. Brain MRI showed mild thinning of corpus callosum. Physical examination at genetic clinic showed head circumference at 3th percentile with body weight and body height at 10–25th percentile. There was facial dysmorphism, namely, hypertelorism, medial flared eyebrows, mild overhanging columella, pointed chin, and fleshy and uplifted earlobes (Figure 1). Based on facial gestalt, Mowat-Wilson Syndrome rather than AS was suspected. ZEB2 gene study was performed. It showed a de novo pathogenic frameshift mutation* ZEB2*{NM_014795.2}:c.[3335delACTT];[=];p.*ZEB2*{NP_055610.1}:p.[Tyr1112Cysfs^*∗*^128][=]. Thus the diagnosis of Mowat-Wilson Syndrome was substantiated.

### 2.4. Case  4

A 2-year-old girl was referred from developmental paediatrician for developmental delay with AS phenotype, namely, flat occiput and wide mouth. She was the first child of the nonconsanguineous Chinese couple, born at full term with birth weight of 2.9 kg. The perinatal history was unremarkable. She had hypotonia and feeding difficulties during early infancy. Assessment at 1 year and 6 months showed that she had moderate to severe grade developmental delay with autistic features. Baseline investigations included brain MRI and metabolic screening was normal. There was no seizure, regression, or stereotypical hand movement. However, she had occasional abnormal outburst of laughter. The head size was normal at 10–25th percentile. Despite the fact that she had some behavioral features of AS, overall clinical profile was not typical (Figure 1). Therefore array CGH was performed, which showed a de novo arr[Hg18] 22q13.31q13.33(45,355,784-49,522,658)x1. That means that a terminal deletion in chromosome 22 at band q13.31 region with the size of 4.17 Mb included the* SHANK3* gene; thus the diagnosis of Phelan-McDermid Syndrome was substantiated.

## 3. Summary and Conclusion

Loss of maternal inherited* UBE3A* gene predominantly expressed in the brain was the pathomechanism of AS. Only 90% of clinically diagnosed AS would have identifiable molecular defect. The remaining 10% were labeled as test negative Angelman-like Syndrome. These Angelman-like Syndromes are actually separate disease entities that are not the variations of AS. However, due to overlapping clinical phenotype, their differentiation is sometimes challenging. Over the last decade, there were many novel AS mimic diseases being discovered and summarized in Table 1 [6–8]. The molecular basis for those AS mimic diseases could also be classified into two emerging classes, namely, the chromatin-remodeling disorder and synaptopathies [6]. However, there were still many of them with uncertain mechanism that had clinical phenotypes overlapping with AS.

The characteristic facial gestalt of AS included microcephaly, flat occiput, divergent squint, wide mouth, and widely spaced teeth. Given the phenotypic overlapping between the AS and Angelman-like Syndrome, clinical differentiation was difficult. Despite this, there were some distinct features that could be useful for clinical diagnosis and guided the further genetic testing. In case 1, the diagnosis was* FOXG1* related congenital variant of Rett Syndrome. It was first reported in the literature in 2011 [9]. The core clinical features of* FOXG1* related disease included early onset postnatal microcephaly, severe mental retardation, hypotonia, absent speech, dyskinesia, and corpus callosum hypogenesis [10, 11]. The other reported MRI brain abnormalities included delayed myelination and gyral simplification [11]. In this case, the early onset of postnatal microcephaly together with hypoplasia of corpus callosum was suggestive of* FOXG1* related disease. Epilepsy was also common but relatively easy to control as compared with* CDKL5* related disorder [11], another Angelman-like Syndrome. Distinct EEG pattern like high voltage slow delta activity and intermittent high-amplitude rhythmic theta activity would occasionally differentiate the AS from other Angelman-like Syndromes [8].

In case 2, the diagnosis was Rett Syndrome due to* MECP2* mutation. It was well reported that Rett Syndrome and AS have overlapping clinical features including seizures, impaired sleep pattern, inappropriate laughter, and ataxia [12]. However, normal period of development during at least first 6 months of life followed by developmental regression was quite distinctive for Rett Syndrome. Unless the epilepsy was poorly controlled, regression was unusual for AS. Although it was reported that AS had particular EEG pattern, there was also specific pattern in Rett Syndrome like generalized background slowing and/or loss of occipital dominant rhythm, with further theta and delta slowing as the developmental regression continued [12, 13].

In case 3, the diagnosis was Mowat-Wilson Syndrome due to loss of function in* ZEB2* gene on chromosome 2q22.3. The features resembling AS included moderate to severe grade intellectual disability, happy predisposition, epilepsy, and microcephaly [14]. However, congenital structural anomalies including Hirschsprung disease, congenital heart disease, and corpus callosum hypoplasia were far more common in Mowat-Wilson Syndrome than in AS. The most distinguished feature was the facial gestalt including hypertelorism, telecanthus, medial flared eyebrow, uplifted earlobes with central depression, overhanging nasal tip, low inserted columella, and prognathism [15]. It was well known that not all these facial features were present during early life and diagnosis could be missed during early childhood.

The diagnosis in case 4 was Phelan-McDermid Syndrome (PMS). It was the first microdeletion syndrome that was reported to mimic AS [16, 17]. The shared clinical features included moderate to severe grade global delay with absent speech, hypotonia, and neonatal feeding difficulties that happened in our case, but mild overgrowth with large hands, large ears, and dysplastic toenails would be the distinctive features for PMS [17, 18]. Posterior cranial fossa brain malformations were also well reported in PMS but not in AS. This case illustrated that many microdeletion/microduplication syndromes were masqueraded Angelman-like Syndrome that array CGH should be the first investigation for them.

**Figure 2:**
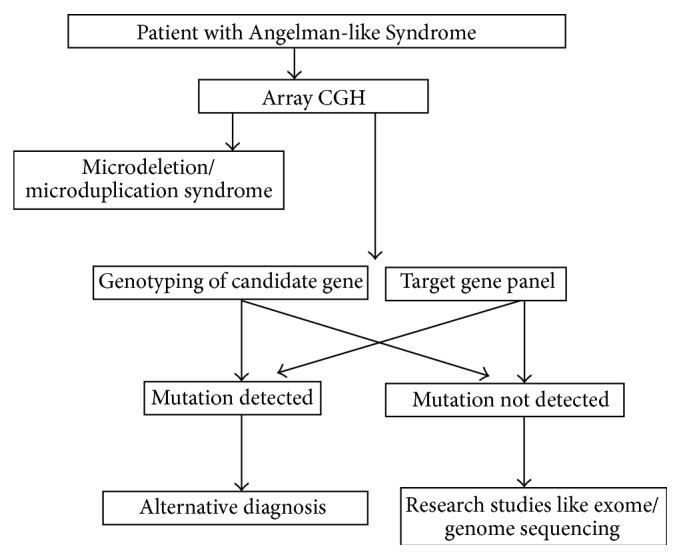
The genetic diagnostic algorithm of Angelman-like Syndrome.

The clinical features of selected Angelman-like Syndrome were summarized in Table 2. In terms of genetic testing for Angelman-like Syndrome, two categories of diseases based on the genetic mechanisms should be considered. These included microdeletion/microduplication syndrome and single gene syndrome. Therefore, after methylation study and* UBE3A* gene analysis, the first line of investigation for Angelman-like Syndrome should be array CGH. If negative, either single gene analysis based on clinical phenotype or targeted gene panel by next generation sequencing should be pursued. The proposed diagnostic algorithm for Angelman-like Syndrome was depicted in Figure 2.

In conclusion, the Angelman-like Syndrome was not uncommon. With the advancement of genomic testing, many emerging diseases have been identified with AS mimic phenotype. Accurate diagnosis is important as the pathogenesis, potential treatment, prognosis, and mode of inheritance among them are different. Recognition of distinct features among Angelman-like Syndrome would provide useful clue in diagnostic strategies. With the jurious use of new technologies like array CGH and next generation sequencing method, it is expected that more and more test negative Angelman-like Syndromes would have definite molecular diagnosis.

## Figures and Tables

**Table 1:** Angelman-like Syndrome.

Chromatin-remodeling disorder		Synaptopathies		Unknown mechanism	
Syndrome	Genes	Syndrome	Genes	Syndrome	Genes
Rett Syndrome/MECP2 duplication syndrome	*MECP2*	Phelan-McDermid Syndrome/22q13.3 deletion syndrome	*SHANK3*	Pitt-Hopkins Syndrome	*TCF4*
Mowat-Wilson Syndrome	*ZEB2*			Christianson Syndrome	*SLC9A6*
Kleefstra Syndrome/9q34.3 deletion syndrome	*EHMT1*			HERC2 deficiency	*HERC2*
*MBD5* haploinsufficiency/2q23.1 deletion syndrome	*MBD5*			Adenylosuccinase deficiency	*ADSL*
Koolen-de Vries Syndrome/17q23.31 deletion syndrome	*KANSL1*			*CDKL5* syndrome	*CDKL5*
Congenital variant of Rett Syndrome	*FOXG1*			*MEF2C* haploinsufficiency syndrome	*MEF2C*
Alpha-thalassemia/intellectual disability syndrome	*ATRX*			Ohtahara Syndrome	*STXBP1*
				Methylenetetrahydrofolate deficiency	*MTHFR*

**Table 2:** Differentiating clinical features among Angelman-like Syndromes.

	AS	Rett	MWS	FOXG1	KS	PMS	PHS	CS	CDKL5	MEF2C	ARTX
Microcephaly	**+**	**+**	**+**	**+**	**+**		**+**	**+**	**+**	**+**	**+**
Seizure	**+**	**+**	**+**	**+**	**+**		**+**	**+**	**+**	**+**	
Speech impairment	**+**	**+**	**+**	**+**	**+**	**+**	**+**	**+**		**+**	**+**
Ataxia	**+**	**+**					**+**	**+**		**+**	
Stereotypical hand movements	**+/**−	**+**		**+**			**+**		**+**	**+**	
Tremulous/jerky limb movements	**+**										
Happy predisposition	**+**	**+**	**+**				**+**				**+**
Abnormal MRI			**+**	**+**		**+**	**+**	**+**			
Hyperventilation/apnea episode		**+**					**+**				
Sleep disturbances	**+**	**+**		**+**	**+**	**+**					
Hirschsprung disease			**+**								
Lack of purposeful hand use		**+**									
Prominent jaw/chin	**+**		**+**								
Wide mouth	**+**		**+**				**+**				
Upturned ear lobes			**+**								
Genital anomalies					**+**						**+**
Congenital heart disease			**+**		**+**						**+**
Developmental regression		**+**				**+**		**+**			
Others		In female only				Mild overgrowth	Persistent finger pad Constipation	In male only			In male only HbH in blood smear

AS: Angelman Syndrome; MWS: Mowat-Wilson Syndrome; KS: Kleefstra Syndrome; PMS: Phelan-McDermid Syndrome; PHS: Pitt-Hopkins Syndrome; CS: Christianson Syndrome; ARTX: alpha-thalassemia/intellectual disability syndrome.
